# Hybrid Proton-Exchange Membrane Based on Perfluorosulfonated Polymers and Resorcinol–Formaldehyde Hydrogel

**DOI:** 10.3390/polym13234123

**Published:** 2021-11-26

**Authors:** Alexandra Maria Isabel Trefilov, Adriana Balan, Ioan Stamatin

**Affiliations:** 1 National R&D Institute for Laser, Plasma and Radiation Physics (INFLPR), 077125 Bucharest-Măgurele, Romania; alexandra.trefilov@inflpr.ro; 2 Faculty of Physics, University of Bucharest, 3NanoSAE Research Center, 077125 Bucharest-Măgurele, Romania

**Keywords:** cation-exchange membrane, resorcinol–formaldehyde polymer gels, fuel cell, perflourosulfonated acid membrane, ionic conductivity

## Abstract

Organic resorcinol–formaldehyde (RF) hydrogels were introduced into a hybrid cation-exchange membrane in order to enhance its following properties: water uptake, thermal stability, and ionic conductivity. This study was aimed to investigate the modifications induced by the RF organic clusters that form a uniform distributed network within the perflourosulfonated acid (PFSA) matrix. RF concentration was controlled by resorcinol and formaldehyde impregnation time using water or ethanol solvents. The specific morphological and structural properties were characterized by atomic force microscopy, UV–Vis, and Fourier transform infrared spectroscopy. Thermo-gravimetric analysis was employed to study the thermal stability and degradation processes of the composite membranes. Proton conductivity, as a function of relative humidity (RH) at 80 °C, was measured using in-plane four-point characterization technique. Compared to the pristine membrane, the PFSA–RF hybrid membranes showed improved thermal stability at up to 46 °C and higher ionic conductivity for low RF content, especially at low relative humidity, when using ethanol-based solvents. Single fuel cell testing on RF-based membrane–electrode assembly revealed impeccable fuel crossover and power performance at 80 °C and 40% relative humidity, delivering a 76% increase in power density compared to a reference assembled with a pristine membrane and the same catalyst loadings.

## 1. Introduction

Conventional energy sources using fossil fuels are the main environmental pollution cause. Switching to renewable energy sources such as hydro, wind, or solar energy seems to be the natural choice. However, most of these sources are variable, unpredictable, and highly dependent on geographical position [1,2]. In this context, electrochemical systems are attracting attention due to their advantages, such as high energy density, low emission of pollutants, fast response times, and facile scalability. In particular, polymer electrolyte membrane fuel cells (PEMFCs) are alternative electrochemical power sources developed for a wide area of applications in various areas, such as portable and stationary power generation, transport, military, and space electric devices [3,4,5,6]. Despite technological progress, the prohibitive cost and durability of the high-performance materials represent the main barriers to wider commercialization. Recently, a flagship in PEMFC was introduced: the aerostack [7], which is a new concept in water-management technology that when combined with a self-humidifier membrane and high-performance lightweight materials can raise the power density up to 1 W g^−1^. This shows that there is enough room for improvement such as new filtration membranes for low hydrogen purity or new engineered perfluorosulfonic acid (PFSA) membranes with more than one sulfonic group on the structural repeat unit that can improve ionic conductivity. The targets for PEM based on PFSA membranes imposed by national projects (US-DOE and Horizon 2020) are to achieve ionic conductivities higher than 0.1 S cm^−1^ at 80 °C and 50% RH and to devise catalysts less affected by the CO-poisoning.

Today, the best known proton conductor is PFSA, which consist of hydrophobic polytetrafluoroethylene (PTFE) backbone and perfluorinated side chains, terminating with hydrophilic sulfonic acid groups (–SO_3_H). The pendant sulfonic side groups during hydration develop spherical ionic clusters with inverted micellar structure [8]. The ionic clusters are interconnected by narrow channels forming a cluster network with high ionic selectivity strongly dependent on the hydration level [9]. This is the archetype structure presented in the earlier studies to explain the ionic transport through perfluorosulfonated ionomers related to the water swollen morphology respective the percolation pathways to reach maximum ionic conductivity [10,11]. The ratio, mole perfluorinated sulfonic groups/mole PTFE (equivalent weight, EW) determine the thermal and mechanical stability, ion-exchange capacity (IEC = 1000/Me), water uptake, the proton conductivity [12,13,14].

**Figure 1:**
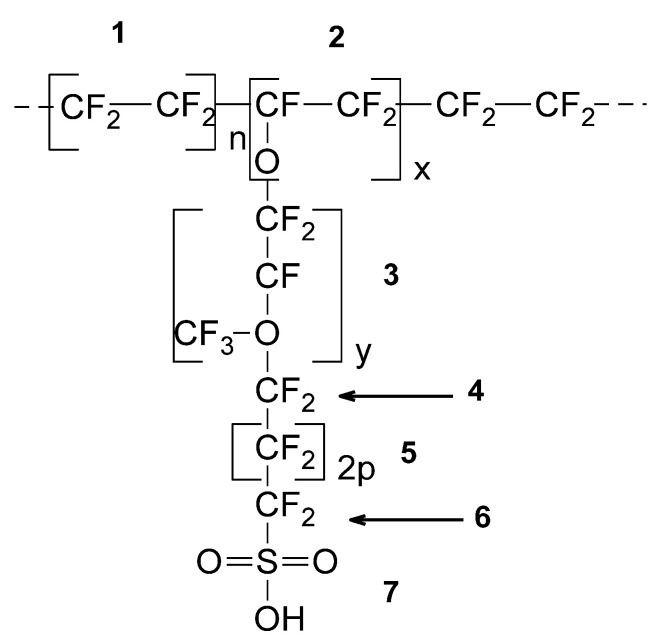
Structural repeating unit (SRU) for PFSA/polytetrafluoroethylene (PTFE) copolymers.

The basic characteristics of PFSA membranes can be summarized with the structure–properties relationship using the Van der Waals approach developed for polymers [15,16]. The basic structure consists of a copolymer backbone of tetrafluoroethylene units and perfluorinated sulfonic acid (Figure 1). The Van der Waals approach estimates the basic characteristics: equivalent weight and the polymer volume occupied in 3D space, free volume space, and the coefficient of molecular packing (Table 1) [17].

The most known and studied membranes among the commercial representatives are Nafion (Du Pont), Flemion (Dow Chemicals), Fumion (FumaTech) [18]. Until now, molecular engineering and polymer synthesis have not produced an advanced ionomer with physical and chemical properties better than those of Nafion. In addition, a PFSA with an equivalent weight (EW) greater than 1100 g mole^−1^ has not been reported in the literature, although a range of up to 105–106 Da has been achieved. These ionomers present excellent proton conductivity when well hydrated, with a maximum proton conductivity of 0.2–0.05 S cm^−1^ at 30 °C, depending on equivalent weight (i.e., the conductivity of Nafion with EW 1100 is 0.1 S cm^−1^).

However, despite all the favorable properties, at high operation temperatures (above 100 °C) or at low humidity, a PFSA membrane is easily dehydrated, which leads to a sharp decrease in proton conductivity, membrane shrinkage, and, subsequently, the mechanical degradation of the membrane–catalyst interface [19]. Membrane behavior has a high impact on fuel cell performances. When used in vehicles, fuel cells are operated in harsh conditions: open-circuit voltage (OCV) holding, idling, dynamic load, startup–shutdown, full-power running, overload, and freezing–thawing [20].

In this respect, the goal of molecular and nanocomposite engineering is to develop new improved proton-exchange membranes better than the only commercial product currently available, Nafion. Research has been focused on three main directions: (1) obtaining a proton conductivity higher than 110 mS cm^−1^ and an ion-exchange capacity >1 meq g^−1^; (2) achieving self-humidification with/without additives to avoid fast dehydration with a decrease of proton conductivity when operating above 100 °C; and (3) attaining improved durability and stability in terms of fuel crossover, chemical properties, and thermo–mechanical properties [13,21]. Various materials with high proton conductivity, good water retention capacity, and reinforcing agents—such as silicon oxides, expanded polytetrafluoroethylene (e-PTFE) fibrils, polyvinylidene fluoride, and other polymers—have been used [22,23,24].

The latest research has considered a wide range of approaches in producing composite membranes that include copolymerization, ionic–nonionic polymer blends, and inorganic–organic composite membranes with silica, phosphates [25], ionic liquids [26], conductive, and electro-active polymers [14,27,28].

The main issue encountered in inorganic–organic hybrid membrane synthesis is the occurrence of hygroscopic filler aggregation when applying the recasting procedure. This phenomenon limits the contact between fillers and Nafion matrix. Y. Choi et al. [19] reported the synthesis of a hybrid membrane with a uniform filler dispersion in a Nafion membrane using Nafion hydrophilic ionic clusters as nano-scale reactors to synthesize sulfonated RF polymer gels in the ionic clusters. The membrane electrode assembly (MEA) fabricated with 2 wt.% sulfonated RF polymer gel/Nafion hybrid membrane showed a maximum power density of 289 mW cm^−2^ without humidification. However, the pristine Nafion membrane showed better performance when reactant gases were fully humidified.

We propose a novel impregnation method for obtaining RF-modified PFSA membranes. Pristine perfluorinated membranes are immersed in a resorcinol and a p-toluene sulfonic acid aqueous solution followed by a polycondensation reaction when exposed to a formaldehyde solution. The composite membranes were obtained via the in situ sol–gel process into membrane pores, where resin concentration was controlled by resorcinol and formaldehyde impregnation time using water or ethanol solvents. Given the stability and the high conductivity of the RF resin, the goal was to improve ionomer membrane properties. We further describe the synthesis procedure and present the results obtained regarding structural analysis, thermal behavior, and proton conductivity.

## 2. Materials and Methods

### 2.1. Materials

PFSA Membrane: grade Fumapem 1050 (supplier FuMA-Tech GmbH, Bietigheim-Bissingen, Germania) with the following technical specifications: equivalent weight EW of 1000 g/eq, specific conductivity in acidic form of >85 mS cm^−1^, water uptake of 25 wt.% at room temperature, thickness of 50–60 microns, dimensional swelling in water at 80 °C, 7%, density of 1.98–2 g cm^−1^, glass transition temperature of 110 °C, and temperature of start thermal decomposition of 270–300 °C.

Fumapem, F-1050, based on the technical data specifications, is a PFSA/PTFE copolymer similar in structure to Nafion [29]: -[(CF-CF2)-(CF2-CF2)x]y-, where x is ~6, as estimated from equivalent weight (EW = 1000 g/eq).

RF precursors: Resorcinol (R) and p-toluene sulfonic acid (PTSA) water/ethanol solutions (R/PTSA = 5) and a 37% aqueous formaldehyde solution. Reagents were purchased from Sigma Aldrich at grades for chemical analysis.

### 2.2. Preparation of Membranes with RF Resin

Membrane activation. Fumapem F1050, in salt form, was treated in a 10% aqueous solution of HNO_3_ for 3 h at 90 °C. After washing, the membranes were boiled and thermally treated with demineralized water for 1 h at 90 °C. Finally the membranes were washed with demineralized water (~pH 7) and kept in a 0.5 M H_2_SO_4_ solution. Before immersion in a resorcinol solution, F-1050 was dried at 80–85 °C and is hereon referred to as S0.

PFSA–RF composite. Modified membranes were obtained by exposing S0 reference membranes to a resorcinol (R) and p-toluenesulfonic acid (PTSA) solution with an R:PTSA molar ratio of 5:1 (using demineralized water or ethanol as the solvent) for different time intervals (1, 10, and 30 min), followed by immersion in a formaldehyde solution for the same time intervals. Finally, the samples were washed with distilled water in order to remove by-products and dried in air at 50 °C for 1 week with gradual humidity loss. Sulfonic acid groups in the PSFA membrane act as a driving force, and resorcinol molecules are absorbed in the polar environment. Then, upon the addition of formaldehyde, typical sol–gel reactions (addition and poly-condensation) occur within membrane pores. Moreover, PTSA acts as an acidic booster, accelerating the polymerization reaction, with the advantage over the basic catalysts that it prevents the incorporation of alkaline metal impurities [30,31]. Regarding the solvents, one can note that alcohol opens up membrane pores better than water, facilitating the gelation process. The downside of ethanol is that it may react with formaldehyde, generating hemiacetal and acetal molecules that do not react with resorcinol [32,33,34].

Notations. Samples are referred to as follows: SW1, SW10, and W30 (obtained in water-based resorcinol solutions for different immersion time intervals) and SE1, SE10, SE30 (obtained in ethanol-based resorcinol solutions for different immersion time intervals).

### 2.3. Membrane–Electrode Assembly Preparation

Membrane–electrode assemblies were produced based on a previously documented procedure [35]. The gas diffusion layer was composed of a 3 × 3 cm Toray Carbon Paper (TGP-H-120, 5% Wet Proofing) backing layer and a microporous layer (MPL) with 0.3 mg cm^−2^ carbon loading. The MPL was composed of 90 wt.% commercial plasma-pyrolyzed carbon black (PL-CB13, PlasmaChem, 550 m^2^ g^−1^; 13 nm average particle size) and 10 wt.% PTFE (Teflon PTFE DISP 30, Chemours|DuPont™, FuelCellStore), corresponding to the minimum PTFE loading needed to balance power performance and water flow management. The carbon black and PTFE resin were dispersed in isopropanol, ultrasonicated with an ultrasonic liquid processor for 10 min at 80 W cm^−2^ of sonication intensity for ink homogenization, and uniformly spray-coated on the carbon paper backing layer.

The catalyst layer was fabricated by uniformly spraying, on the GDL surface, the catalyst ink that consists of Pt/C powder (Alfa Aesar, 60% Pt) and a 15% Nafion solution (5% Nafion Solution in alcohol, Dupont^®^). The Pt/C and Nafion were initially mixed with a few drops of ultrapure water and then dispersed in isopropanol (analytical grade, 99%, Merck). The slurry was ultrasonicated with an ultrasonic liquid processor for 5 min at 80 W cm^−2^ of sonication intensity for ink homogenization, uniformly spray-coated on the GDL, and then dried at 75 °C for 12 h. The catalyst loadings were 0.3 mg/cm^2^ for the anodes and 0.6 mg/cm^2^ for the cathodes. MEAs were obtained by hot-pressing against the membranes at 125 °C and 0.8 kN cm^−2^ of pressure for 15 min. Samples are referred to as follows: MEA_S0 and MEA_SE1, signifying those containing the pristine and SE1 membranes, respectively.

### 2.4. Analytical Techniques

Topography and phase contrast images were obtained by means of SPM-NTegra Prima AFM (NT-MDT Spectrum Instruments, Moscow, Russia), operated in semi-contact mode using an NSG 01 cantilever (resonance frequency: 87–230 kHz; force constant: 1.45–15.1 N m^−1^). Images were processed and analyzed by means of the offline NT-MDT Image Analysis 2 software.

Ultraviolet–visible (UV–vis) spectroscopy (Spectrophotometer UV–Vis, V-570, JASCO International Co., Ltd., Sennincho Hachioji, Japan) was used to obtain the absorbance spectra of the modified membranes in the 200–800 nm wavelength interval, with a 1 nm resolution.

FT-IR Spectroscopy was performed with an FT-IR Jasco Spectrometer, model 6200 (JASCO International Co., Ltd., Sennincho Hachioji, Japan) in the range 4000–400 cm^−1^, resolution 0.4 cm^−1^.

For the RF concentration, PFSA membrane was weighted before ($m_{\mathrm{PFSA}}$) and after ($m_{\mathrm{PFSA}-\mathrm{RF}}$) the impregnation procedure after drying at 80 °C for 12 h, and the RF content was determined by the following relationship:

(1) $$\mathrm{RF}\left[\%\right]=\frac{m_{\mathrm{PFSA}-\mathrm{RF}}-m_{\mathrm{PFSA}}}{m_{\mathrm{PFSA}}}\cdot 100$$

Water uptake (WU) was calculated by comparing the weight of each membrane sample in the wet and in the dry state using the following equation:

(2) $$\mathrm{WU}\left[\%\right]=\frac{m_{\mathrm{wet}}-m_{\mathrm{dry}}}{m_{\mathrm{dry}}}\cdot 100$$

The dry membrane weight, $m_{\mathrm{dry}}$, was determined after drying the sample in the oven for 12 h at 80 °C. The sample was then soaked in ultrapure water for 24 h. The wet membrane weight, $m_{\mathrm{wet}}$, was obtained after drying the surface with filter paper and then immediately weighing the sample [36].

Ion-exchange capacity (IEC) was determined as follows: acidic-form membranes were converted to salt0form membranes via immersion in 1 M NaCl solutions for 24 h; the exchanged H^+^ ions in solutions were titrated with 0.05 M NaOH solutions in the presence of phenolphthalein:

(3) $$\mathrm{IEC}\left[\mathrm{meq}/g\right]=\frac{V_{\mathrm{NaOH}}\left[\mathrm{mL}\right]\cdot C_{M\;\mathrm{NaOH}}}{m_{\mathrm{dry}}}$$

The number of water molecules per sulfonic acid fixed site ($\lambda_{w}$) was determined by the following formula:

(4) $$\lambda_{w}=\frac{\left(m_{\mathrm{wet}}-m_{\mathrm{dry}}\right)/M_{H_{2}O}}{\mathrm{IEC}\times m_{\mathrm{dry}}}$$

where $M_{H_{2}O}=18.01{\;g\;\mathrm{mol}}^{-1}$ is water molecular weight and $m_{\mathrm{wet}}$ the weight of the membrane after soaking in water for 24 h.

Thermogravimetric analysis (TGA/SDTA Mettler Toledo, Greifensee, Switzerland) was performed in air at a heating rate of 10 °C/min in the temperature range of 25–400 °C. Before measurements, all membranes were conditioned at RT for 24 h. Broido’s method [37] was used for the evaluation of the non-isothermal kinetic parameters from the TG data. The activation energy for thermo-oxidative degradation was estimated using the following equation:

(5) $$\ln\left(\ln\left(\frac{1}{Y}\right)\right)=-\frac{E_{a}}{\mathrm{RT}}+C$$

where Y is the fraction of the sample not yet decomposed, $E_{a}$ is activation energy of the reaction, R is the universal gas constant, and T is the temperature. The plot ln(ln(1/Y)) versus 1000/T resulted in a straight line with a slope of $-E_{a}/R$.

Ionic conductivity was measured via four-point BekkTech conductivity test cell (BT-512 Membrane Conductivity Test System, Bekktech LLC, Loveland, CO, USA) at a set point temperature of 80 °C. Membrane samples were cut into strips of approximately 15 mm length and 4–5 mm in width before being placed in the four-point probe cell (distance between middle electrodes: 4.2 mm) with temperature and humidity (±1 degree absolute accuracy) controlled in nitrogen gas by a back-pressure regulator. Conductivity testing is a good solution because contact resistance and catalyst effect are eliminated when a membrane electrode assembly is measured.

MEA single-cell testing was performed on a BekkTech BT-512 single cell fuel cell test station (BekkTech LLC, Loveland, CO, USA) controlled by an Agilent 6060 B system, providing precise control over gas temperature, pressure, humidity, and flow rates, as well as the cell temperature. The set operating conditions were as follows: 80 °C cell temperature, 40%/80% relative humidity (RH), 200 SCCM hydrogen flow rate at the anode, and 800 SCCM air flow rate at the cathode.

Prior to MEA performance evaluation, a set of separate electrochemical experiments were carried out in the same configuration as the fuel cell testing: cyclic voltammetry for MEA activation and linear sweep voltammetry (LSV) for hydrogen fuel crossover testing. All these experiments were performed on an OrigaFlex OGF500 potentiostat/galvanostat system (OrigaLys ElectroChem SAS, ±5 nA to ±500 mA current range, ±15 V applied voltage) according to the standard procedures described in our previous studies [38].

MEA activation procedure, performed by high-frequency cyclic voltammetry, promoted membrane hydration, promoted catalyst recovery, and cleared the pathways needed by the reactants to reach the active sites of the catalyst layer, thus ensuring high cell performance.

Hydrogen crossover, carried out to assess MEA degradation and membrane integrity, was performed in the same temperature and humidity conditions as the integrated MEA testing. The cathode, supplied with 200 SCCM of humidified nitrogen, served as the working electrode (WE), while the anode, supplied with 200 SCCM of hydrogen, served as both the counter and reference electrode (CE/RE). LSV plots were performed at a scan rate of 4 mV s^−1^ in the voltage interval of 0.1–0.8 V vs. CE/RE. The measured current density indicates the amount of hydrogen crossing over from the anode to the cathode. The hydrogen crossover flux, *C_H_* (mol cm^−2^ s^−1^), was calculated according to the following Faraday’s equation:

(6) $$C_{H}=\frac{J_{lim}}{nF}$$

where *J_lim_* (mA cm^−2^) is the limiting current density, obtained at the cathode from the asymptotic LSV value at the 0.3 V mark; *n* is the number of exchanged electrons in the reaction (*n* = 2); and *F* is the Faraday constant (96,485.3329 C mol^−1^).

## 3. Results and Discussion

### 3.1. Morphological Properties

**Figure 2:**
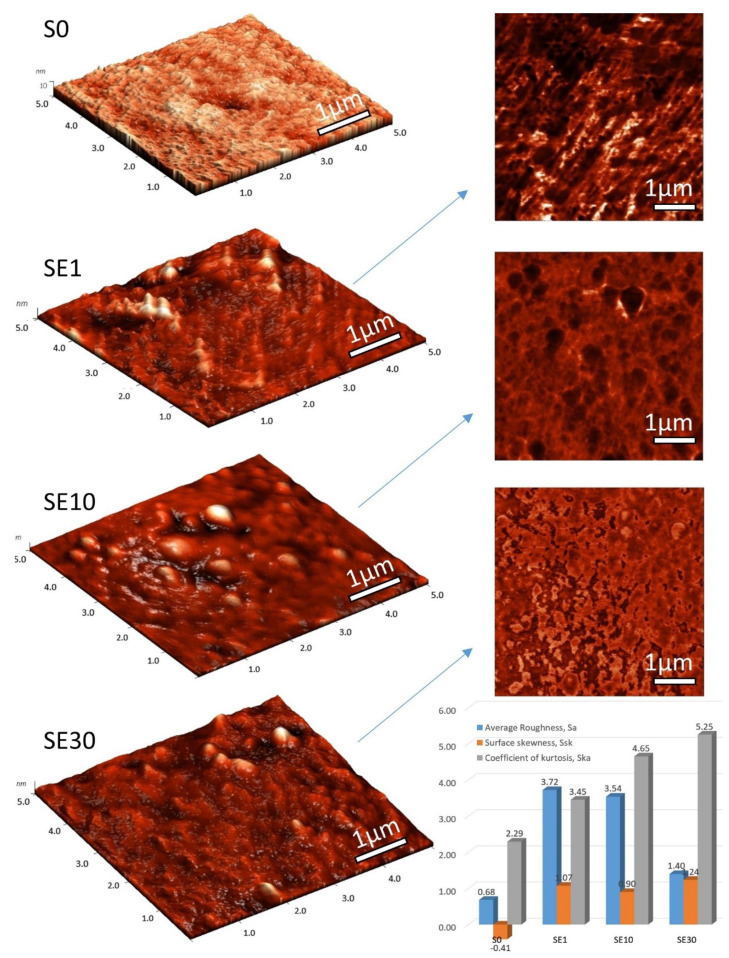
AFM images of S0 Ref unmodified membrane and PFSA–RF samples using an ethanol–water mixture as the solvent in the impregnation process: 3D images and the corresponding phase contrast images on 5 µm * 5 µm scan areas. Graph representing average roughness (nm), surface skewness, and coefficient of kurtosis resulted from the data-processing of the AFM image.

We took AFM images of the S0 unmodified membrane and PFSA–RF samples using an ethanol–water mixture as the solvent in the impregnation process; 3D images and the corresponding phase contrast images on 5 µm × 5 µm scan areas are shown in Figure 2. The topography reveals significant morphological differences on membrane surfaces with increasing RF content. The globular structures of the organized polymeric chains in the pristine membrane were crowded by the RF polymer structures in the composite membranes. According to Gierke, a pristine membrane is an aggregation of polymeric chains observed as elongated objects integrated with a continuous ionic medium [10]. RF domains are integrated by condensation within the membrane pores, also modifying surface properties. The average roughness of a pristine membrane is approximately 0.6 nm, very close to uniform polymer membrane reported in the literature [39], but the average roughness of our modified membranes exceeded 3 nm. The exception was the SE30 sample, where the average roughness value was higher than the reference but lower than that of the other samples due to the increased content RF that smoothens the surface (see graph in Figure 2). Analyses of skewness (symmetry of the grain distribution) and the coefficient of kurtosis (describes the sharpness of the probability density of the profile) are shown as well. The coefficient of kurtosis for the pristine membrane was 2.29, meaning that there were relatively few high peaks and low valleys. On the other hand, for the RF membranes, this coefficient was higher than 3, specific for surfaces with relatively many high peaks and low valleys. The skewness parameter also changed depending on the RF content. The skewness parameter was employed for quantitatively characterizing the occasional deep valleys or high peaks, with a value of zero corresponding to the symmetrical height distribution.

Profiles with deep valleys have a negative skewness, whereas high peaks have positive skewness [40]. The skewness parameter for the reference membrane was negative, but once RF was embedded, it reached values in the range of 0.90–1.24. It is likely that the RF structures entered the pores (smoothing the appearance of membrane pores) and also built up on top of the original globular structures (increasing peaks).

### 3.2. Structural Characterization

**Figure 3:**
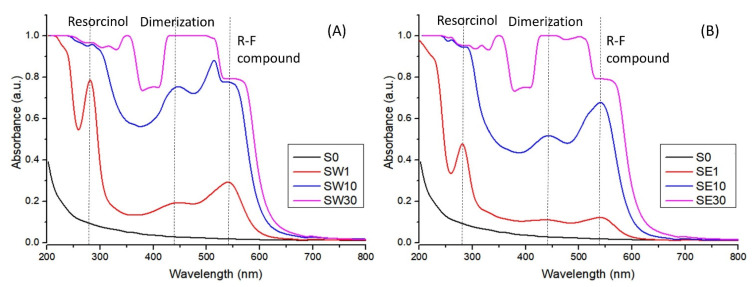
UV–Vis spectra for PFSA and PFSA–RF samples using water (**A**) and an ethanol–water mixture (**B**) as the solvent in the impregnation process.

The UV–Vis-specific features of the RF-modified membranes using water as the solvent in the impregnation process are presented in Figure 3. After immersing the resorcinol-impregnated membranes in the formaldehyde solution, an optical response was produced due to the poly-condensation reaction in the active pores. The acidic functional groups within the perfluorosulfonated membrane along with the PTSA act as catalysts for the poly-condensation reaction and enabled RF gel formation [29].

One could note a shift in the membranes’ color from light purple to reddish brown depending on the time of exposure to the formaldehyde solution, which was also evidenced in the UV–Vis spectra. The absorption increased with the F exposure time and implicitly with RF gel concentration in the membrane. The RF gel resulting from the polycondensation reaction provided the absorption band at 540 nm, related to the charge transfer transitions of the π-conjugated and π-stacked donor–acceptor units [41]. The absorption band at 270 nm appeared due to an aromatic ring in pure resorcinol present in the membrane, while the broad band at 440 nm was specific to the dimerization of resorcinol [42].

**Figure 4:**
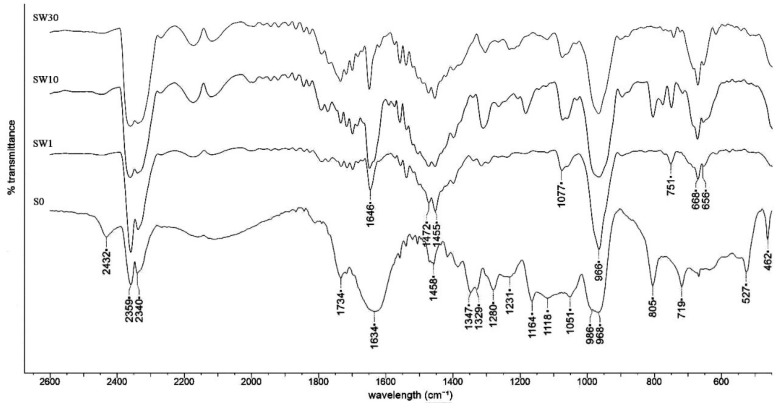
FT-IR spectra for PFSA and PFSA–RF samples using water as the solvent in the impregnation process.

FT-IR-specific features of the RF-modified membranes using water as the solvent in the impregnation process are presented in Figure 4. The perfluorosulfonated membrane spectra analysis shows a typical fingerprint as reported in literature [43,44,45]: 551 cm^−1^ associated with torsion and bending vibrations t (CF_2_); 626–653 cm^−1^ associated with rotation vibrations (CF_2_); 717 cm^−1^ associated with symmetry vibrations (CF2); 805 cm^−1^ associated with (C–S) vibrations; 963–980 cm^−1^ associated with (C–O–C) vibrations; 1059 cm^−1^ associated with symmetry vibrations (SO3-); 1127 cm^−1^ associated with asymmetry vibrations (SO3-); 1150–1243 cm^−1^ associated with asymmetry vibrations (CF2); 1300–1319 cm^−1^ associated with (C–C) vibrations; and 1471 cm-^1^ associated with asymmetry vibrations of (SO3H). In PFSA–RF spectra, an RF-resin-specific response could be identified: 1646 and 1490 cm^−1^ associated with C=C aromatic benzene ring stretching and scissor vibrations, respectively; 1380, 1311, and 1297 cm^−1^ associated with O–H in-plane bending; 1167 and 1152 cm^−1^ associated with C–O symmetric and asymmetric stretching, respectively; 1077 cm^−1^ associated with the C–O–C linkage stretching vibrations of methylene ether bridges between two resorcinol molecules; and 773,740 cm^−1^ associated with C–H aromatic group stretching and out of plane bending [46,47,48,49].

### 3.3. Thermal Behavior of PFSA–RF Resin Systems

**Figure 5:**
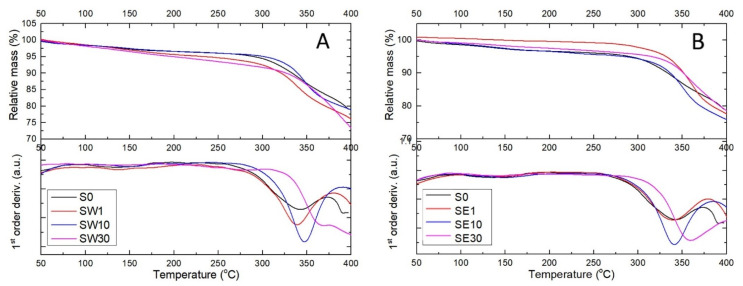
Thermogravimetric analysis of RF-modified membranes using water (**A**) and an ethanol–water mixture (**B**) as the solvents in the impregnation process performed in air at a heating rate of 10 °C/min.

The thermal behavior of PFSA–RF membranes is shown in Figure 5, where a few specific features can be distinguished: the onset temperature of the decomposition reactions, the temperature corresponding to the maximum reaction rate, and the activation energy evaluated using Broido’s method are summarized in Table 2.

A continuous mass loss of up to 5% was observed for all samples within the temperature interval of 25–200 °C due to residual/bounded water loss or/and very weak dehydrogenation of the -HSO_3_ pendant groups. In RF-modified membranes, thermal effects were dependent on the RF–solvent couple and the number of sulfate groups within the membrane matrix. The onset temperature of the thermal degradation of the cross-linked PFSA–RF membrane was found within the interval of 305–340 °C in TGA curves, that is, about 46 °C more than for the reference membrane.

The start temperature of thermal degradation is an indication of the highest processing temperature that can be used. Moreover, the study of the kinetics of the decomposition reactions is key element in the identification of degradation mechanisms. Broido’s method was used to evaluate the activation energy of the thermal degradation processes from the TGA curves (see Table 2).

The activation energy was found to significantly increase with the increasing RF content, from 8.89 to 11.19 kJ mol^−1^ for the SW sample set (water solvent) and from 4.46 to 11.47 kJ mol^−1^ for SE sample set (ethanol solvent). The activation energies for all PFSA–RF samples were considerably lower than the activation energy of the reference sample, i.e., 15.78 kJ mol^−1^. This was an indicator that as the weight loss processes occurred at higher temperatures, the energy barriers decreased. Polymer configuration was strongly influenced by the cross-linking process occurring via the RF polycondensation reaction.

### 3.4. Water Uptake, Hydration Number, and Ion-Exchange Capacity

The RF content, water uptake, ion-exchange capacity, and number of water molecules per sulfonic acid groups for the S0 and PFSA–RF membranes are summarized in Table 3. The RF content was found in the interval of 5.8–43.1% when using water and within the interval of 6.9–68.5% for ethanol solutions (Table 3). The RF content was found to be lower for samples where water was used as precursor solvent rather than ethanol, since the alcohol molecule was bigger than the water molecule. The perfluorinated membrane in acidic-form S0 Ref showed a water content of 28.78%, and the RF-modified membranes’ WU values ranged from 15.28 to 30.21%. At very low RF content, WU values were slightly increasing due to the resin’s high-absorbent characteristic. When high number of cross-linked structures were embedded in the PFSA matrix, pores were partially blocked so the hydration level was reduced. The low WU values for the last two samples translated into a lower number of water molecules per sulfonic acid site. On the other hand, IEC increased for all composite samples due to the resin’s additional sulfonic groups. Although the number of sulfonic acid groups increased, the composite membranes’ IEC raise was low (between 5% and 25%) due to the presence of the sulfonic acid groups isolated within hydrophobic regions of the PFSA matrix, thus making it impossible to contribute to the water retention and proton transfer processes [26].

### 3.5. In-Plane Conductivity

In proton-exchange membranes, proton conductivity is determined by several conduction mechanisms: (a) proton hopping at the surface, (b) Grotthuss diffusion in volume pores, and (c) the bulk diffusion of hydronium ions, where protons travel with water molecules as H_3_O^+^ ions. On the other hand, in the Grotthuss mechanism, a proton passes from one solvent molecule to a neighboring one without bulk diffusion or electro-osmosis [50]. Conduction mechanisms are directly influenced by the number of water molecules within a system [51,52,53]. Hydration water can be found in different states [54]: free water molecules, water molecules strongly or weekly bounded to sulfonic acid groups, and water molecules bounded to the RF resin.

**Figure 6:**
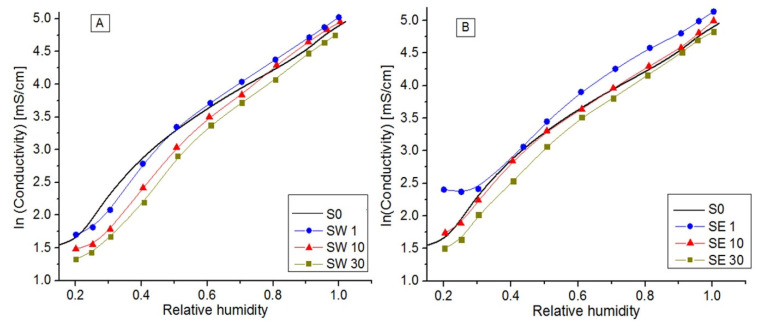
Log plot of the electrical conductivity vs. relative humidity for RF inserted into PFSA using water (**A**) and ethanol–water (**B**) solvents.

**Figure 7:**
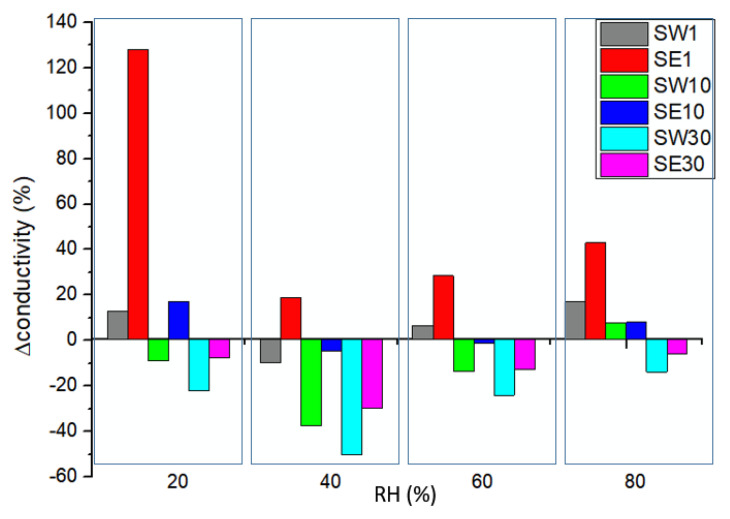
Conductivity variation for the RF-modified PFSA membranes relative to the reference membrane conductivity at different levels of relative humidity.

The ionic conductivity measured at fixed temperature (80 °C) with a specific dependence on relative humidity (RH) for each PFSA–RF–solvent couple is shown in Figure 6. Moreover, the conductivity variation for the RF-modified PFSA membranes relative to the reference membrane conductivity at different levels of relative humidity is clearly evidenced in Figure 7.

PFSA conductivity increased with RH, reaching its maximum value of ~142 mS/cm when fully hydrated. In a log plot function of RH (Figure 6), three distinct regions were evidenced, defined by RH = 30% and RH = 80%, where conduction mechanisms change ($\lambda_{w}$) and the number of water molecules per equivalent of polymer or by the number of sulfonic groups is the determinant parameter in the system at a given volume and temperature [53]. As the hydration level increases, the factors that control the transport phenomena change from cation–sulfonate interactions to water content and its solvation effects [55]. Moreover, $\lambda_{w}$ is strongly dependent on samples’ physical properties, as shown in Table 3. The low-RF-content composite membranes (SW1, SE1, and SE10) presented a higher ionic conductivity than the reference for RH > 50%, while the samples with higher RF contents presented lower ionic conductivity values. This was in accordance with the decrease presented by the IEC and $\lambda_{w}$ values, since a part of the sulfonic acid groups are isolated within hydrophobic regions of PFSA matrix, most likely blocking the conduction paths [29].The Gierke model considers that the water sorption swells the hydrophilic domains and there is a threshold (percolation threshold) amount of adsorbed water at which proton conductivity starts and which increases with the water activity [10]. For a typical PFSA percolative system, a gradual increase in conductivity is produced while increasing the volume fraction of the conductive phase, and then a rapid increase happens in the vicinity of the percolation threshold followed by a further gradual increase until RH = 1. In accordance with these facts, above 80% humidity, the PFSA membrane proton conductivity started to decline, while the low-resin-content membranes presented a higher stability than the PFSA percolation threshold due to the different microstructures created by the RF resin inside the polymer matrix. Nucleophilic aromatic rings served as active sites for ionic complexation and promoted the ionic transport throughout the polymer matrix [56].

The composite membrane obtained using the ethanol solvent presented higher conductivity values than in the case of the membrane synthesized using water solvents. Conduction mechanisms defined by dissociation processes on sulfonic pendant groups and proton transport occurs by hopping or migration, depending on membrane humidification and the RF–solvent–couple.

**Figure 8:**
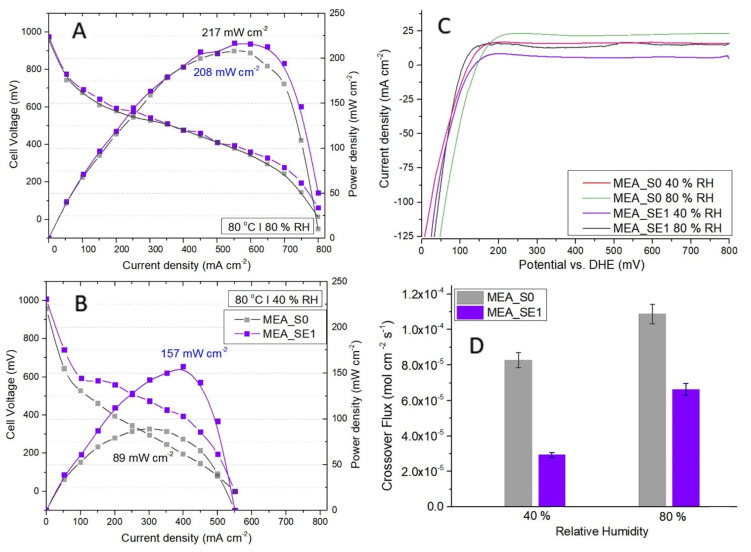
Polarization curves of comparative I–V and power density profiles between the SE1-based and reference MEAs at 80 °C and 80% relative humidity (**A**), respectively, at 80 °C and 40% relative humidity (**B**). Peak power densities given next to the corresponding profiles. In situ polarization testing was performed at 70 kPa of overall pressure in galvanostatic mode by controlling the current and applying fixed flow rates of 200 SCCM of hydrogen at the anode and 800 SCCM of air at the cathode. (**C**) Hydrogen crossover testing by in situ linear sweep voltammetry for S0 and SE1-based MEA at 80 °C and relative humidity values of 40% and 80%; (**D**) hydrogen crossover comparative results for the SE1-based and the reference MEAs over all testing conditions.

Furthermore, since the SE1 sample, i.e., RF-modified membrane using an ethanol-based solvent and 1 min of exposure, proved to behave best in terms of ionic conduction at low relative humidity; single fuel cell testing was carried on using the selected membrane. MEA containing the pristine membrane and the same catalyst loadings was considered as a reference. Before performing polarization measurements, membrane integrity was assessed through in situ hydrogen crossover testing (Figure 8C). Crossover from the anode to the cathode through the membrane is an indicator of pinhole formations. Besides the impact on fuel cell performances, the permeation of reactants through the membrane accelerates degradation processes as peroxide and hydroperoxide radicals are formed [57,58,59]. The current density was determined by the crossover hydrogen consumption rates at the cathode. After 0.3 V, we noted a stabilization of the current due to the termination of hydrogen desorption.

Comparing the LSV analyses of both samples confirmed that the hydrogen crossover fluxes increased with humidity because the induced swelling was affecting the pinhole openings (Figure 8D). Moreover, the increased water content in the membrane may have caused changes in the values of both H_2_ solubility and the diffusion coefficients [59]. Regarding the RF influence on the crossover process, our results proved that the resin improved membrane resistance to hydrogen permeation, both at low and high humidity. The resin acted as “repairing agent” for the possible pinholes in the polymer matrix and blocked or reduced some of the larger pores.

Polarization curves of comparative I–V and power density profiles between the SE1-based and the reference MEAs at 80% and 40% relative humidity are presented in Figure 8A,B. At high humidity, the MEA-SE1 maximum power density of 217 mW cm^−2^ was only slightly higher than the 208 mW cm^−2^ value for the reference due to the hydration level in the membranes increasing proton conductivity. On the other hand, the SE1 membrane showed significantly improved performance in the single-cell PEMFC under low humidity conditions (157 mW cm^−2^ compared to 89 mW cm^−2^). This was consistent with the higher proton conductivity of the RF-modified membranes under low-humidity conditions shown in Figure 7. Resin domains formed in the polymer matrix played an active role in retaining water and preventing the dehydration of the membrane.

## 4. Conclusions

The present study tested the hypothesis of using organic RF gels as dopants in a hybrid cation-exchange membrane in order to increase water uptake, thermal stability, and ionic conductivity. Although the hydration level was reduced as a consequence of the isolation of sulfonic acid groups with hydrophobic regions of the PFSA matrix, the onset temperature of thermal degradation of the PFSA–RF membranes increased by up to 46 °C more than for the reference membrane. Conduction mechanisms in PFSA membranes are defined by dissociation processes of sulfonic pendant groups, and proton transport occurs by hopping or migration depending on membrane humidification and the RF–solvent couple. The ionic conductivity study showed that the low RF content membranes had higher ionic conductivity then the pristine membranes, especially at low relative humidity, when using ethanol-based solvents. Furthermore, the SE1 membrane showed significantly improved performance in the single-cell PEMFC under low humidity conditions, with lower permeation to hydrogen and delivering a 76% increase in power density compared to a reference assembled with a pristine membrane and the same catalyst loadings. Studies are to be continued for the optimization of experimental conditions and the testing of alcohol permeability and conductivity in alcohol-operating conditions for direct methanol/ethanol fuel cells.

## Figures and Tables

**Table 1:** Molecular weight and Van der Waals volume for fragments in SRU.

SRU (See Figure 1)	1	2	3	4	5	6	7
**M_wu_ (g/mol)**	100	97	166	50	50	50	81
**V_VWu_ (Å^3^)**	54.20	49.60	90.40	30.20	27.10	27.98	37.93
Molecular weight copolymer: M_wc_ (x, y, p) = 97x + 166y + 100p + 181 Van der Waals volume: V_wc_ (x, y, p) = 49.6x + 90.4y + 54.2p + 96.11 Molecular weight SRU: M_w_ = 100n + M_wc_ (x, y, p) Van der Waals volume SRU: V_w_ = 54.2n + V_wc_ (x, y, p)							

**Table 2:** Parameters resulted from thermogravimetric analysis.

Sample	t_onset_ (°C) *	t_max_ (°C) **	E_a_ (kJ/mol)
S0	294	338	15.78
SW1	309	337	8.89
SW10	323	347	7.06
SW30	340	386	11.19
SE1	305	336	4.46
SE10	316	339	10.25
SE30	331	355	11.47

* onset temperature of the decomposition reactions; ** temperature corresponding to the maximum reaction rate.

**Table 3:** PFSA–RF-modified membrane characteristics.

Sample	Solvent	Immersion Time (min)	RF (%)	WU (%)	IEC (meq/g)	$\lambda_{w}$
S0	-	0	0.00	28.78	1.05	15
SW1	water	1	0.575	28.42	1.32	12
SW10	water	10	1.650	26.46	1.22	12
SW30	water	30	4.910	15.28	1.14	7
SE1	ethanol	1	0.695	30.22	1.25	13
SE10	ethanol	10	2.033	25.93	1.16	12
SE30	ethanol	30	6.849	20.54	1.11	10

## Data Availability

The data presented in this study are available on request from the corresponding authors.
